# HYPERTENSION SELF-CARE AMONG MEDICARE BENEFICIARIES WITH SELF-REPORTED HEARING TROUBLE

**DOI:** 10.1093/geroni/igad104.3402

**Published:** 2023-12-21

**Authors:** Laura Wallace, Nicholas Reed

**Affiliations:** University of Pennsylvania, Philadelphia, Pennsylvania, United States; Johns Hopkins Bloomberg School of Public Health, Baltimore, Maryland, United States

## Abstract

Hearing loss is common in older adults and frequently comorbid with chronic illnesses like hypertension, which requires self-care practices learned through patient-provider health communication. However, hearing loss is a barrier to communication. This cross-sectional analysis used the 2020 Medicare Current Beneficiary Survey of (N=11,587; weighted estimate, 51.8 million) to characterize hypertension self-care outcomes (measuring blood pressure, taking blood pressure medications, reducing alcohol, and confidence in blood pressure control) by hearing status among Medicare beneficiaries ages >65. In the sample, 39% reported a little and 6% reported a lot of trouble hearing while 66% reported having received a hypertension diagnosis. In models adjusted for sociodemographic and health variables, Medicare beneficiaries with a little trouble hearing (OR=0.84 95% CI=0.72-0.97; P=0.02) and a lot of trouble hearing (OR=0.74; CI 0.57-0.95; P=0.02) had 16% and 26% reduced odds of reporting confidence in hypertension control compared to beneficiaries with no hearing trouble. Similar results were found when excluding hearing aid users (a little trouble hearing OR=0.85; CI=0.72-1.01; P=0.06; a lot of trouble hearing OR=0.69; 95% CI=0.52-0.91; P=0.01). No significant difference was found for other self-care outcomes. To the authors’ knowledge, this is the first characterization of hearing difficulty and hypertension self-care and findings align with previous work suggesting hearing loss may be a barrier to vital health communication. Future work should focus on examining self-care activities across chronic illnesses, temporal relationships, and designing population-appropriate interventions to improve self-care understanding among adults with hearing loss.

